# Combined femoral and acetabular version and synovitis are associated with dGEMRIC scores in people with femoroacetabular impingement (FAI) syndrome

**DOI:** 10.1002/jor.25568

**Published:** 2023-04-12

**Authors:** Nicholas J. Murphy, Jillian Eyles, Libby Spiers, Emily J. Davidson, James M. Linklater, Young Jo Kim, David J. Hunter

**Affiliations:** ^1^ The University of Sydney, Sydney Musculoskeletal Health and the Kolling Institute Faculty of Medicine and Health and the Northern Sydney Local Health District Sydney Australia; ^2^ Department of Orthopaedic Surgery John Hunter Hospital New Lambton Heights Australia; ^3^ Department of Rheumatology Royal North Shore Hospital St Leonards Australia; ^4^ Department of Physiotherapy, Centre for Health, Exercise and Sports Medicine University of Melbourne Melbourne Australia; ^5^ Department of Radiology Royal Prince Alfred Hospital Sydney New South Wales Australia; ^6^ Castlereagh Imaging St Leonards St Leonards Australia; ^7^ Department of Orthopedic Surgery Boston Children's Hospital Boston Massachusetts USA

**Keywords:** arthroscopy, cartilage, hip, osteoarthritis

## Abstract

This study sought to explore, in people with symptoms, signs and imaging findings of femoroacetabular impingement (FAI syndrome): (1) whether more severe labral damage, synovitis, bone marrow lesions, or subchondral cysts assessed on magnetic resonance imaging (MRI) were associated with poorer cartilage health, and (2) whether abnormal femoral, acetabular, and/or combined femoral and acetabular versions were associated with poorer cartilage health. This cross‐sectional study used baseline data from the 50 participants with FAI syndrome in the Australian FASHIoN trial (ACTRN12615001177549) with available dGEMRIC scans. Cartilage health was measured using delayed gadolinium‐enhanced MRI of cartilage (dGEMRIC) score sampled at the chondrolabral junction on three midsagittal slices, at one acetabular and one femoral head region of interest on each slice, and MRI features were assessed using the Hip Osteoarthritis MRI Score. Analyses were adjusted for alpha angle and body mass index, which are known to affect dGEMRIC score. Linear regression assessed the relationship with the dGEMRIC score of (i) selected MRI features, and (ii) femoral, acetabular, and combined femoral and acetabular versions. Hips with more severe synovitis had worse dGEMRIC scores (partial *η* ^2^ = 0.167, *p* = 0.020), whereas other MRI features were not associated. A lower combined femoral and acetabular version was associated with a better dGEMRIC score (partial *η* ^2^ = 0.164, *p* = 0.021), whereas isolated measures of femoral and acetabular version were not associated. In conclusion, worse synovitis was associated with poorer cartilage health, suggesting synovium and cartilage may be linked to the pathogenesis of FAI syndrome. A lower combined femoral and acetabular version appears to be protective of cartilage health at the chondrolabral junction.

## 1 INTRODUCTION

Femoroacetabular impingement (FAI) syndrome is a condition whereby abnormal motion‐related interaction between the proximal femur and acetabulum produces hip symptoms, increasing the risk of early‐onset osteoarthritis.^1^ Delayed gadolinium‐enhanced MRI of cartilage (dGEMRIC) is a useful imaging technique that enables the detection of early biochemical cartilage alterations before the onset of overt structural damage.^2^ Specifically, dGEMRIC quantifies cartilage glycosaminoglycan concentration by measuring T1 relaxation time after the equilibration of an anionic contrast agent. Worse dGEMRIC indices have been associated with future osteoarthritis development^3^, ^4^ and joint arthroplasty,^4^ suggesting dGEMRIC may help predict subsequent disease progression.

The alpha angle is the bony morphology measurement most strongly linked to the dGEMRIC score and development of subsequent hip osteoarthritis in FAI syndrome.^5^, ^6^, ^7^ However, there is increasing awareness that the femoral and acetabular versions also play a role in the pathomechanics of FAI syndrome.^8^, ^9^ Femoral and acetabular version abnormalities are highly prevalent in FAI syndrome,^10^, ^11^ and have been associated with more severe hip osteoarthritis,^12^ larger labral tears,^13^ higher peak hip joint contact pressures,^14^ and reduced hip range of motion.^15^ Relative femoral retroversion was associated with inferior outcomes from hip arthroscopy in a study of 243 patients undergoing hip arthroscopy for FAI syndrome,^16^ although subsequent studies did not reproduce this finding.^17^ The location of impingement is also affected by the femoral and acetabular versions. People with FAI syndrome and femoral retroversion impinge earlier with maximal flexion, at a more anterior region of the femoral head–neck, compared to others with FAI syndrome and asymptomatic controls.^18^ People with FAI syndrome and acetabular retroversion have a higher occurrence of extraarticular subspine impingement.^19^

There is emerging evidence of the relevance of the combined femoral and acetabular version, the summation of the femoral version, and the central acetabular version (originally coined the McKibbin index^20^) to FAI syndrome.^21^, ^22^ In a series of 200 hips with FAI syndrome, the combined femoral and acetabular version was associated with an internal and external hip range of motion,^23^ whereas femoral and acetabular versions considered in isolation were not. Despite the potential importance of the femoral version, acetabular version, and combined femoral and acetabular version to FAI syndrome, their relationship to early joint damage in FAI syndrome has not been investigated. The measurement of cartilage health using dGEMRIC is a validated and reliable tool for assessing very early joint damage in preosteoarthritic FAI syndrome patients and thus is an appropriate outcome measure for this purpose.

Abnormal bony hip morphology has an established association with poor cartilage health^7^, ^24^; however, cartilage health may also be independently affected by the integrity of tissues such as the synovium, labrum, and subchondral bone. Synovitis is increasingly thought to be important to the pathogenesis of early osteoarthritis,^25^, ^26^ as is subchondral bone remodeling, which may manifest as bone marrow lesions or subchondral cysts.^27^, ^28^ Labral tears have been implicated in hip microinstability and consequent chondrolabral damage.^29^ The relationship of magnetic resonance imaging (MRI)‐delineated soft tissue damage with biochemical cartilage composition, measured by dGEMRIC, has not been studied in FAI syndrome to our knowledge.

This study sought to explore, in patients with FAI syndrome: (1) whether more severe labral damage, synovitis, bone marrow lesions, or subchondral cysts assessed using the Hip Osteoarthritis MRI Score (HOAMS)^30^ were associated with worse dGEMRIC score, and (2) whether abnormal femoral version, acetabular version, and/or combined femoral and acetabular version were associated with worse dGEMRIC score. The chondrolabral junction was selected for dGEMRIC score measurement due to the established importance of cartilage in this region for early osteoarthritic change in FAI syndrome.^31^, ^32^, ^33^ We hypothesized that (1) more severe labral tears and effusion–synovitis would be associated with worse dGEMRIC scores, and (2) abnormal combined femoral and acetabular versions would be associated with worse dGEMRIC scores.

## 2 MATERIAL AND METHODS

### 2.1 Participants

This cross‐sectional study used data collected at baseline from all participants in the Australian FASHIoN trial (Australian Clinical Trials Registration Number: ACTRN12615001177549).^34^ Participants were recruited between February 2015 and January 2018, through 10 different public and private clinics in Australia. FAI syndrome was diagnosed by one of eight orthopedic surgeons experienced in hip arthroscopy. Inclusion criteria were: age ≥16 years, hip pain, cam and/or pincer morphology on imaging (alpha angle >55° and/or lateral center edge angle > 40° or other radiographic sign of pincer morphology), and the treating surgeon believing the patient would benefit from arthroscopic surgery. Exclusion criteria were: pre‐existing osteoarthritis (Tonnis grade >1 or <2 mm joint space width on pelvic radiograph), previous significant hip pathology, injury, or shape‐changing surgery. On entry to the study, participants recorded demographic data and underwent standardized plain radiographs and MRI scans, including dGEMRIC. In the event where participants had bilateral FAI syndrome, they were asked to nominate the most painful hip for entry to the study, and the less painful hip was not included. There were 50 hips from 50 participants included in this study. Of the 99 participants recruited for the Australian FASHIoN trial, only 50 participants had baseline dGEMRIC scans available for analysis in the present study. Only participants that underwent a dGEMRIC scan with Dotarem were included in the present study, whereas participants that underwent a scan with a different gadolinium‐based contrast agent (GBCA) were excluded. Concerns regarding potential deposition in the brain for certain GBCAs^35^ led to a change from Magnevist (GdDTPA; Berlex Labs) to Dotarem (GdDOTA; Guerbet) during the trial.

### 2.2 Imaging protocol

Imaging acquired upon entry into the trial were utilized in this study, including standardized MRI scans with dGEMRIC and plain radiographs. MRI scans included in this study were acquired on two 3 T scanners (Siemens Prisma, MIA Radiology,  or Siemens Skyra, Castlereagh Radiology). Participants received an intravenous injection of GBCA, 0.2 mmol/kg bodyweight of Dotarem, and then walked for 15 min, after which MRI scanning occurred. The dGEMRIC sequences were acquired 45–60 min postcontrast injection. The MRI scan included dGEMRIC sequences (spin‐echo inversion recovery [IR] with fat suppression; sagittal orientation, repetition time [TR] 2340 ms, echo time [TE] 15 ms, slice thickness/slice gap 3.0 mm/3.0 mm, echo train length 11, field of view [FOV] 16 cm × 16 cm, matrix size 256 × 256, number of signal averages 1; 6 IR delays at 50, 100, 200, 400, 800, and 1600 ms), and a sagittal three‐dimensional T2‐weighted true fast imaging with steady‐state precession sequence to be reconstructed for bony morphology measurement (TR 10.2 ms, TE 4.3 ms, slice thickness 0.63 mm, FOV 16 cm × 16 cm, matrix size 256 × 256, number of signal averages 1). Participants also underwent MRI sequences to enable reading of the HOAMS, and axial knee sequences to facilitate femoral version measurement. The comprehensive details of these sequences have been described previously.^34^

### 2.3 Imaging analyses

**Figure 1:**
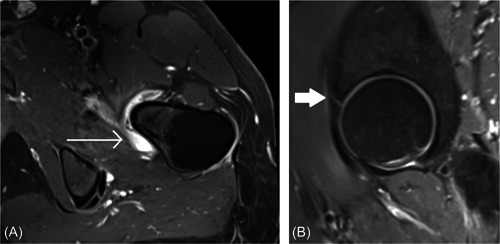
Hip osteoarthritis MRI score features. (A) Axial T1‐weighted fat‐suppressed sequence demonstrating grade 2 synovitis (thin arrow). (B) A sagittal proton density‐weighted fat‐suppressed sequence demonstrating a grade 2 labral tear (thick arrow). MRI, magnetic resonance imaging.

Scoring of the HOAMS was performed by a musculoskeletal radiologist (E. J. D.) after a consensus scoring exercise with a fellow musculoskeletal radiologist (J. M. L.). Weighted kappa scores for inter‐ and intraobserver reliability (Supporting Information: Table 1) were calculated based on 15 scans scored separately approximately 2 weeks later. MRI features were scored for severity of damage at specified subregions as described in the HOAMS validation manuscript.^30^ Features chosen for analysis in this study for possible association with dGEMRIC score were the labrum, subchondral cysts, bone marrow lesions, and effusion–synovitis (see Figure 1). As described in the HOAMS validation manuscript,^30^ effusion–synovitis was scored according to the thickness of contrast‐enhancing fluid signal at four different locations (medial and lateral scored on the coronal plane, and anterior and posterior scored on the axial plane). A thickness of <2 mm was considered grade 0 (normal), a thickness of 2–4 mm as grade 1, and thickness of >4 mm as grade 2 effusion–synovitis^30^ (Figure 1). Effusion–synovitis was measured on the T1‐weighted contrast‐enhanced sequence for this study, rather than synovial inflammation thickening, due to the MRI scanning protocol occurring after 15 min of walking postcontrast injection to facilitate dGEMRIC. Other features scored in the original HOAMS manuscript were not analyzed in this study, as they were not relevant to this cohort in which people with osteoarthritis greater than Tonnis grade 1 were excluded, or because they were thought less likely to be independently associated with cartilage health (dGEMRIC score) after adjustment for the alpha angle and body mass index (BMI).

**Figure 2:**
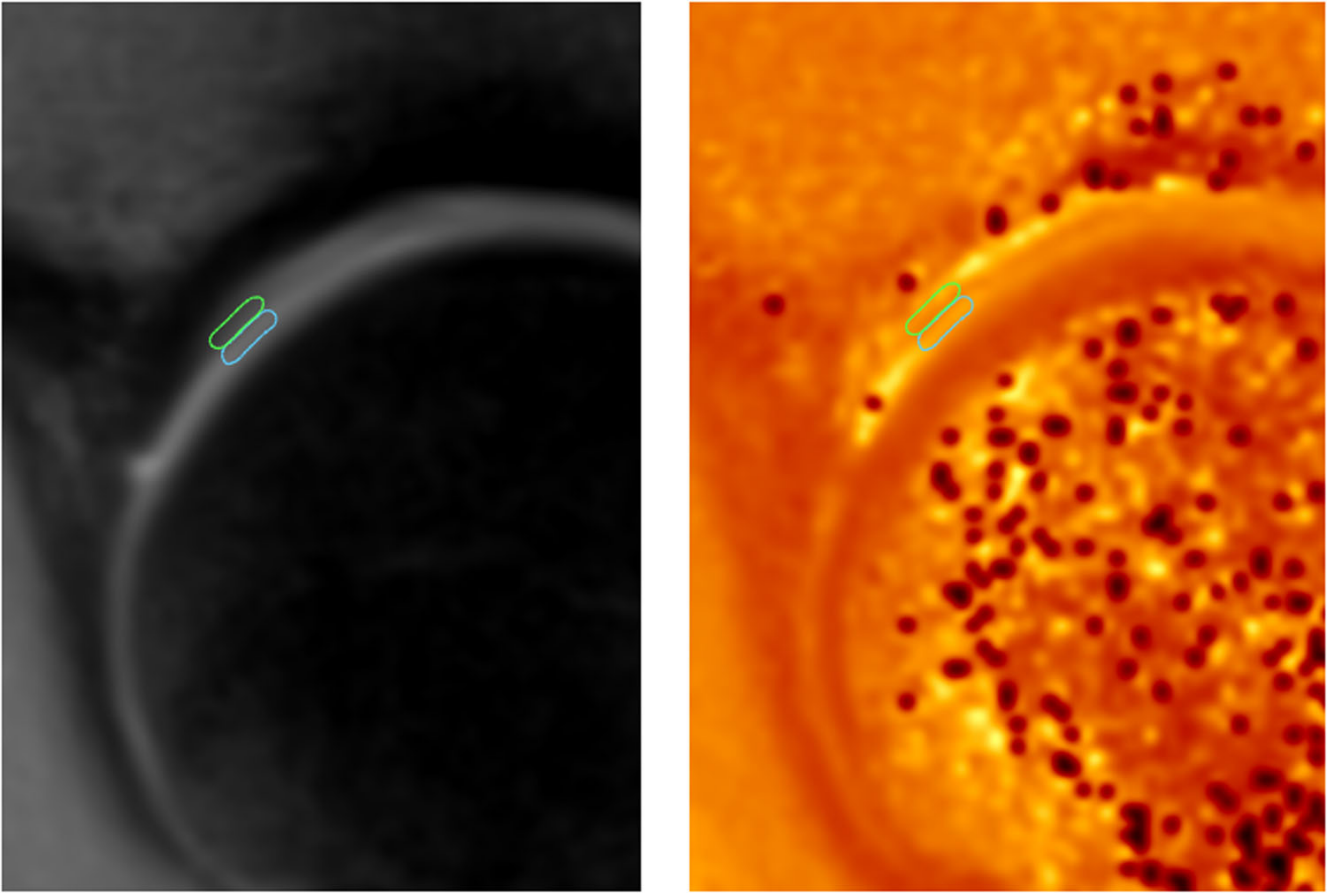
Region of interest (ROI) definition at the chondrolabral junction for delayed gadolinium‐enhanced MRI of cartilage (dGEMRIC): The dGEMRIC analysis was conducted by defining acetabular and femoral head cartilage ROIs at the chondrolabral junction, reaching approximately 3–6 mm toward the acetabular fossa in each hip, on three midsagittal plane slices. MRI, magnetic resonance imaging.

The dGEMRIC analysis was conducted by defining one acetabular and one femoral head cartilage region of interest (ROI) at the chondrolabral junction on each of the three midsagittal slices (i.e., six ROIs in total for each hip), reaching approximately 3–6 mm toward the acetabular fossa in each hip, on three midsagittal plane slices, as described previously^36^ (see Figure 2). Care was taken to define ROIs including only acetabular and femoral head cartilage separately, excluding labrum and bone. Accurate positioning of the midsagittal plane was ensured using a three‐dimensional view of the hip volume in Osirix (version 8).^37^ The ROI definition occurred on the TI 1600 ms IR images, which were co‐registered to the T1 maps. The dGEMRIC analysis was conducted by a trained orthopedic resident (N. J. M.) and the ROI definition was checked by an orthopedic surgeon with approximately 2 decades of experience in dGEMRIC measurement (Y. J. K.). The reproducibility of dGEMRIC measurements in the hip has been established in a prior study,^38^ however inter‐ and intrarater reliability were not measured for this study.

**Figure 3:**
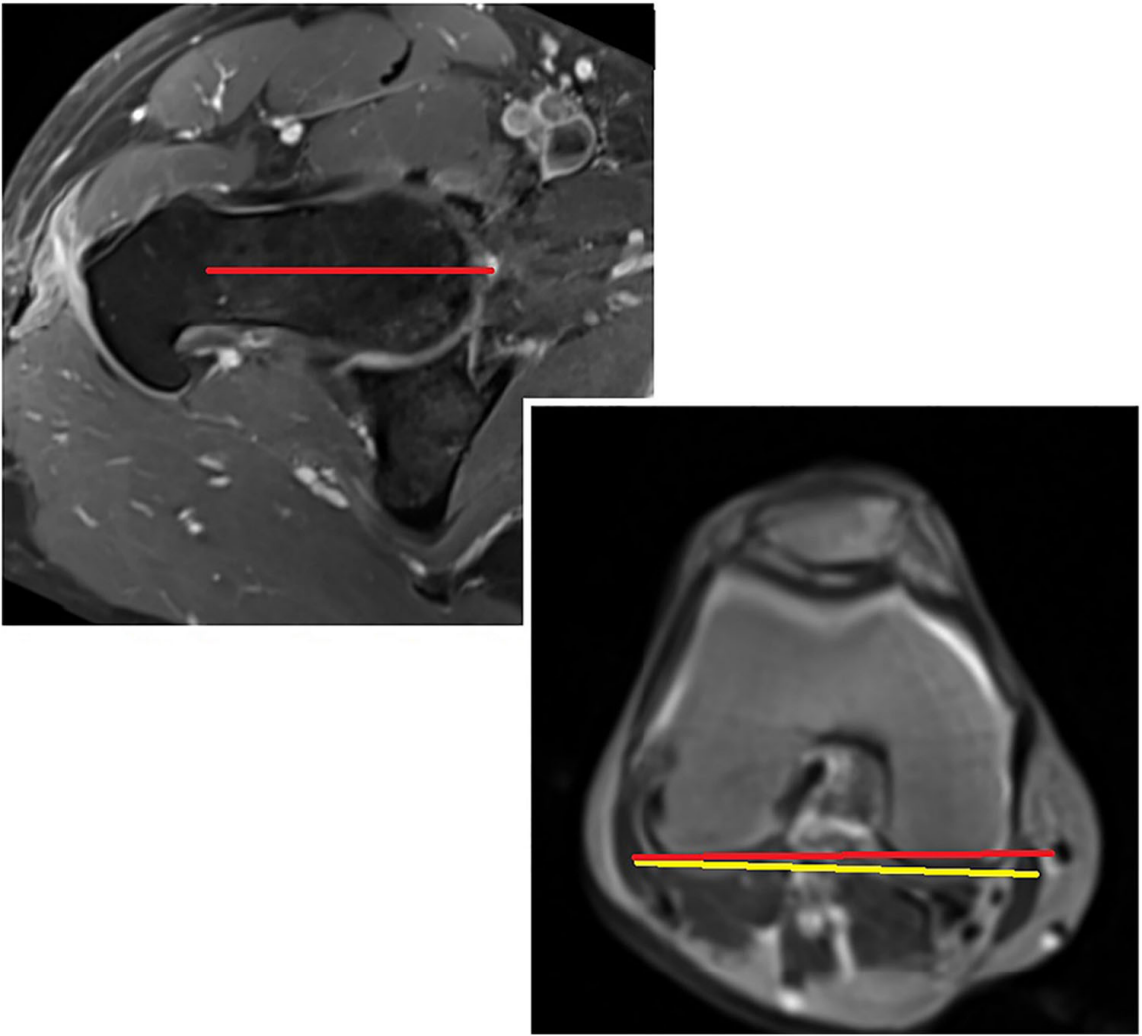
Femoral version measurement. Performed on axial hip and knee sequences using the method described by Reikerals et al.,^40^ whereby a line connecting the femoral head center with the femoral neck center is drawn on an image where the anterior and posterior cortices run parallel to one another The angle this line makes with a line between the posterior condyles of the knee is considered the femoral version. This hip demonstrates a low femoral version.

**Figure 4:**
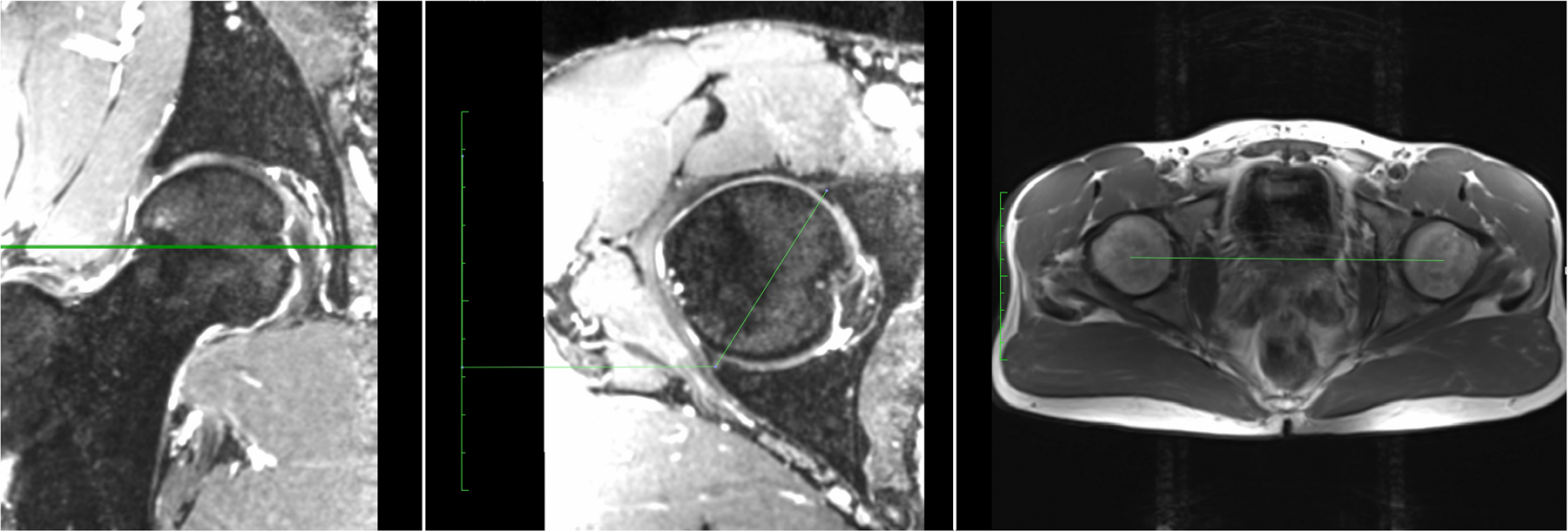
Acetabular version measurement. The acetabular version was measured using axial hip MRI sequences at the level of the center of the femoral head, with the line connecting the center of both femoral heads used as a reference point. MRI, magnetic resonance imaging.

The alpha angle was measured on reconstructed radial planes at four different clock positions at 30° intervals, as originally described by Klenke et al.^39^ The alpha angle was measured at 12 o'clock (superior), 1 o'clock (superoanterior), 2 o'clock (anterosuperior), and 3 o'clock (anterior) using the Orthopaedic Studio OsiriX plugin Version 1.3.3b (Carl Siversson; Lund University). The femoral version was measured using axial hip and knee MRI sequences using the method originally described by Reikerals et al.^40^ (see Figure 3). The acetabular version was measured using axial hip MRI sequences at the level of the center of the femoral head^41^ (see Figure 4). Measurements were performed by a trained orthopedic resident (N. J. M.) after measurement of intra‐ and interrater reliability with another trained reader, which has been published previously.^42^

### 2.4 Statistical analyses

Univariate linear modeling was used to assess the association of the maximum severity score for each nominated HOAMS feature with the dGEMRIC score. The association was tested by adjusting for BMI, alpha angle, and MRI scanner (given there were two different MRI scanners used for dGEMRIC scanning in this study) as these are known to potentially influence the dGEMRIC score.^7^, ^24^, ^43^, ^44^

Univariate linear modeling assessed version measurements for their association with the dGEMRIC score, adjusting for the effects of the alpha angle, BMI, and scanner used. The femoral and acetabular version were each categorized as low (<10°), normal (10–25°), or high (>25°), using the thresholds originally described by Tonnis and Heinecke.^45^ Combined femoral and acetabular version was similarly defined as low (<20°), normal (20–50°), or high (>50°) using the thresholds recently used in the literature.^10^ A sensitivity analysis was also performed using a threshold of 20° for the “high” femoral and acetabular version, which has been used in some prior studies,^11^ and 40° for the “high” combined femoral and acetabular version. To our knowledge, the distribution of combined femoral and acetabular version values in the general population is unknown and thus the upper threshold of 50° used in most studies^21^, ^22^, ^45^ is somewhat arbitrary. This threshold was presumably derived by adding together the commonly used thresholds of 25° for the upper limits of the normal femoral version and the acetabular version. However, this may not be appropriate, as in normal hip joints it has been shown that a complementary relationship exists between the femoral and the acetabular version, whereby a hip with decreased femoral version usually compensates for this with increased acetabular version, and vice versa.^46^

Because there was expected to be substantial multicollinearity between alpha angles measured in the various radial planes (including the maximum alpha angle), only the most significant alpha angle (i.e., lowest *p* value) was used in the multivariable model. Alpha angle also had the potential to be associated with other independent variables in the models, including the femoral version,^47^ or with any of the MRI features being tested for their association with the dGEMRIC score. Because of this, all analyses were also performed without adjustment for alpha angle and the results were inspected to ensure no change in the direction of associations was occurring with the addition of alpha angle to the models due to it exerting a confounding influence. Bonferroni adjustment for multiple comparisons was employed with *α* set at 0.05 and adjusted according to the number of pairwise comparisons for each variable analyzed. The homoscedasticity assumption was assessed using the modified Breusch–Pagan test. Robust heteroscedastic standard errors were used in the case heteroscedasticity was present. All statistical analyses were conducted using Statistical Package for the Social Sciences, version 24 (IBM).

## 3 RESULTS

A summary of participants' demographic details, dGEMRIC scores, bony morphology measurements, and HOAMS is reported in Table 1. The 50 hips included in the study from 50 different participants (age 32.4 ± 11.2 years, 58% males) had a mean dGEMRIC score of 673.6 ms (SD 121.8 ms). Supporting Information: Table 2 reports the univariate association of the alpha angle in each radial plane with the dGEMRIC score. The alpha angle at the 3 o'clock (anterior) position was strongly associated with the dGEMRIC score (*B* = −3.88, 95% confidence interval [CI]: −6.49, −1.27; *p* = 0.004), whereas the alpha angle in other radial planes, including the maximum alpha angle, was not associated. Consequently, the alpha angle at the 3 o'clock (anterior) position was used for adjustment in subsequent models. Adjusted univariate regression analyses for MRI features associated with the dGEMRIC score are reported in Table 2. Effusion–synovitis severity was the only MRI feature significantly associated with the dGEMRIC score. Grade 2 effusion–synovitis, the highest grade of severity, was associated with significantly worse dGEMRIC scores compared to other grades (mean difference grades 0–2 = 141.5, 95% CI: 2.97, 280.09, partial *η* ^2^ = 0.167, *p* = 0.020). Adjusted univariate linear regression analyses for version measurements associated with the dGEMRIC score are reported in Table 3.

**Table 1:** Patient demographic, delayed gadolinium‐enhanced MRI of cartilage (dGEMRIC) data, and hip osteoarthritis score data (*n* = 50).

Patient demographics	
Age (years)	32.4 (11.2)
Sex, *n* (%)	
Male	19 (38%)
Female	31 (62%)
Body mass index (kg/m^2^)	24.3 (3.0)
Type of FAI syndrome, *n* (%)	
Cam	33 (66%)
Pincer	9 (18%)
Mixed	8 (18%)
dGEMRIC score	
Combined acetabular + femoral ROIs (ms)	673.6 (121.8)
Acetabular ROI (ms)	649.7 (132.4)
Femoral ROI (ms)	698.7 (130.2)
Bony morphology measurements	
Superior alpha angle (12 o'clock)	53.4 (16.5)
Superoanterior alpha angle (1 o'clock)	63.6 (14.5)
Anterosuperior alpha angle (2 o'clock)	63.2 (14.6)
Anterior alpha angle (3 o'clock)	57.0 (12.6)
Maximum alpha angle (all radial planes)	70.9 (12.6)
Femoral version, *n* (%)	
Low (<10°)	17 (34%)
Normal (10–25°)	26 (52%)
High (>25°)	7 (14%)
Acetabular version, *n* (%)	
Low (<10°)	4 (8%)
Normal (10–25°)	44 (88%)
High (>25°)	2 (4%)
Combined femoral and acetabular version, *n* (%)	
Low (<20°)	11 (22%)
Normal (20–50°)	37 (74%)
High (>50°)	2 (4%)

**MRI feature**	**Maximum severity grade**	**Count**
Synovitis, *n* (%)	0	12 (24%)
	1	33 (66%)
	2	5 (10%)
Subchondral cyst, *n* (%)	0	30 (60%)
	1	17 (34%)
	2	3 (6%)
	3	0
Labral injury, *n* (%)	0	1 (2%)
	1	3 (6%)
	2	40 (80%)
	3	6 (12%)
Bone marrow lesion, *n* (%)	0	47 (94%)
	1	2 (4%)
	2	1 (2%)
	3	0

*Note*: Values are mean (standard deviation) unless otherwise stated.

Abbreviations: CI, confidence interval; FAI, femoroacetabular impingement; MRI, magnetic resonance imaging; ROI, region of interest.

**Table 2:** Univariate association of nominated hip osteoarthritis MRI score features with delayed gadolinium‐enhanced MRI score.^a^

	Mean difference ~(*M*_d_)	95% CI of *M*_d_	Partial *η* ^2^	*p* Value
Synovitis			**0.167**	**0.020**
Grades 0–1	−7.88	−94.50, 78.74		1.000
Grades 0–2	141.50	2.97, 280.09		0.044
Grades 1–2	149.40	21.35, 277.47		0.017
Subchondral cysts			**0.039**	**0.420**
Grades 0–1	−21.47	−104.51, 61.59		1.000
Grades 0–2	67.02	−98.02, 232.06		0.952
Grades 1–2	88.48	−80.83, 257.80		0.600
Labral injury			**0.015**	**0.890**
Grades 0–1	−54.55	−427.80, 318.70		1.000
Grades 0–2	−14.50	−331.84, 302.84		1.000
Grades 0–3	8.39	−347.64, 364.42		1.000
Grades 1–2	40.05	−152.05, 232.16		1.000
Grades 1–3	62.94	−160.75, 286.63		1.000
Grades 2–3	22.89	−131.35, 177.13		1.000
Bone marrow lesions			**0.063**	**0.245**
Grades 0–1	133.41	−138.69, 405.52		0.686
Grades 0–2	134.99	−143.84, 413.81		0.703
Grades 1–2	1.58	−385.28, 388.43		1.000

*Note*: Bold values indicate overall partial *η* ^2^ and *p* value for each MRI feature.

Abbreviations: CI, confidence interval; MRI, magnetic resonance imaging.

^a^ Adjusted for alpha angle and body mass index, with Bonferroni adjustment for multiple comparisons

**Table 3:** Pairwise comparison between low, normal, and high version measurements with respect to delayed gadolinium‐enhanced MRI score.^a^

	Mean difference ∼(*M*_d_)	95% CI of *M*_d_	Partial *η* ^2^	*p* Value
Femoral version			**0.104**	**0.095**
Low (<10°) to normal (10–25°)	85.36	−15.69 to 186.40		0.124
Low (<10°) to high (>25°)	18.53	−104.79 to 141.84		1.000
Normal (10–25°) to high (>25°)	−66.84	−182.42 to 48.75		0.471
Acetabular version			**0.109**	**0.083**
Low (<10°) to normal (10–25°)	61.05	−83.44 to 205.33		0.895
Low (<10°) to high (>25°)	210.44	−20.09 to 440.96		0.084
Normal (10–25°) to high (>25°)	149.39	−39.27 to 338.04		0.165
Combined femoral and acetabular version			**0.164**	**0.021**
Low (<20°) to normal (20–50°)	104.77	9.12 to 200.41		0.028
Low (<20°) to high (>50°)	2.90	−191.7 to 197.52		1.000
Normal (20–50°) to high (>50°)	−101.87	−285.75 to 82.02		0.524

*Note*: Bold values indicate overall partial *η* ^2^ and *p* value for each version category.

Abbreviations: CI, confidence interval; MRI, magnetic resonance imaging.

^a^ Adjusted for alpha angle and body mass index, with Bonferroni adjustment for multiple comparisons.

Low (<20°) combined femoral and acetabular version was associated with a significantly better dGEMRIC score compared to hips with normal (20–50°) combined femoral and acetabular version (mean difference low–normal = 104.77, 95% CI: 9.12, 200.41, partial *η* ^2^ = 0.164, *p* = 0.021), but femoral and acetabular version considered in isolation were not significantly associated. Sensitivity analyses modeling the association of version measurements and MRI features with dGEMRIC scores, without adjustment for alpha angle, are shown in Supporting Information: Table 3. The finding for the combined femoral and acetabular versions remained the same without correction for the alpha angle, however low femoral version became statistically significant in its association with better dGEMRIC score (partial *η* ^2^ = 0.216, *p* = 0.005) and the acetabular version approached statistical significance (partial *η* ^2^ = 0.126, *p* = 0.051). The sensitivity analysis using a threshold of 20° for high femoral and acetabular versions and 40° for high combined femoral and acetabular versions produced the same findings as the primary analysis (Supporting Information: Tables 4a, 4b).

## 4 DISCUSSION

The most significant findings of this cross‐sectional study were that (1) severe effusion–synovitis was associated with a worse dGEMRIC score at the chondrolabral junction, and (2) low combined femoral and acetabular version was associated with a better dGEMRIC score at the chondrolabral junction, compared to normal or high combined femoral and acetabular version. These associations were independent of the effect of alpha angle and BMI, which were already known to influence the dGEMRIC score.

There is an expanding body of research that implicates synovitis in the progression of osteoarthritis.^26^, ^48^ The presence of synovitis and cartilage lesions have been particularly closely linked,^49^, ^50^ although studies aiming to determine which of these is the antecedent cause have been inconsistent in their findings. In both osteoarthritic and non‐osteoarthritic knees, synovitis was found to predict the subsequent development of cartilage lesions.^49^, ^51^ Conversely, a study on hips in the general population^50^ found that the presence of cartilage lesions predicted subsequent effusion–synovitis. The proposed mechanisms underlying this relationship include the inflamed synovium releasing multiple inflammatory mediators that induce cartilage degradation, as well as cartilage fragments themselves inducing synoviocytes to behave in an inflammatory fashion.^48^ Being cross‐sectional in design, the present study does not provide insight into which effusion–synovitis or cartilage lesions may be the “chicken or egg” in early hip osteoarthritis. However, it demonstrates that an association exists between effusion–synovitis and the early‐stage biochemical cartilage deterioration detected by dGEMRIC. The relationship between synovitis and cartilage damage has not been previously investigated in patients with FAI syndrome to our knowledge. Given that dGEMRIC is not commonly performed in clinical practice, the presence of effusion–synovitis may be a surrogate marker of potential early cartilage damage that is worth the radiologist bearing in mind when assessing articular cartilage in FAI syndrome patients.

The absence of a significant association with the dGEMRIC score for other HOAMS features (labral tears, bone marrow lesions, and subchondral cysts) bears mentioning. Bone marrow lesions and subchondral cysts have been previously associated with cartilage lesions in osteoarthritis.^52^, ^53^ It is possible that the relationship between these MRI features and biochemical cartilage deterioration may not be established until later in the disease course, and hence was not detected in this FAI syndrome cohort. This was an exploratory study not purposefully powered for these analyses, and certain features such as bone marrow lesions were uncommon in this young, non‐osteoarthritic cohort, therefore it is also possible that associations of more modest strength were not detected in our analyses.

Given that impingement occurs as an interaction of the proximal femur and acetabulum in FAI syndrome, it is intuitive that combined femoral and acetabular versions would have a more easily detectable association with cartilage health than either femoral or acetabular versions considered in isolation. However, the direction of association found in this study, lower combined femoral and acetabular version being associated with better dGEMRIC score, seems counterintuitive at first glance. The lower combined femoral and acetabular version has been associated with a reduced hip internal rotation range of motion^23^ and is hence more prone to impingement anterosuperiorly with flexion, adduction, and internal rotation. A possible explanation is that lower combined femoral and acetabular anteversion, although increasing the propensity for direct abutment of the femoral head‐neck on the anterosuperior labrum, may cause a “contrecoup” lesion in the posteroinferior region of the acetabulum. This has been described in pincer impingement, whereby persistent anterosuperior abutment of the femur against the acetabular rim causes chronic levering of the femur and microinstability posteroinferiorly.^54^, ^55^ In this scenario, greater shear stresses, which are known to be particularly unfavorable to cartilage metabolism,^56^ are experienced posteroinferiorly. Consistent with our findings, a cadaveric biomechanical study suggested that a tendency to femoral retroversion distributes higher hip contact pressures posteroinferiorly, possibly due to abutment anterosuperiorly producing the described mild levering effect and “contrecoup” damage posteroinferiorly.^14^ Although lower combined femoral and acetabular version may be protective of cartilage at the anterosuperior chondrolabral junction, there may be cartilage injury elsewhere in the joint, such as posteroinferiorly, which this study was unable to detect. The findings in this study may relate to where we measured the dGEMRIC score. Broadly consistent with this study's findings was a meta‐analysis that found higher, rather than smaller, femoral version to be associated with more severe hip osteoarthritis.^12^ Higher femoral version, compared to normal or low femoral version, has also been associated with larger labral tears on hip arthroscopy in hips with FAI syndrome.^13^

To our knowledge, this is the only study that has examined the relationship of MRI features and version measurements to cartilage health using dGEMRIC in FAI syndrome patients. It used validated and reliable imaging methods in the form of the HOAMS^30^ and dGEMRIC.^57^ There are also significant limitations that must be considered. Due to the delay in scanning post‐contrast injection required for dGEMRIC, there was contrast diffusion into the joint in the absence of inflammation, meaning we were unable to distinguish effusion–synovitis from synovial inflammation thickening. Although effusion is highly correlated with synovitis,^25^, ^58^ synovial inflammation‐thickening would be better detected using a nondelayed protocol. The dGEMRIC score was only measured at the chondrolabral junction in this study. Although this is not the first study to sample the dGEMRIC score at this location,^36^ other studies investigating the dGEMRIC score in FAI syndrome have sampled ROIs at different locations,^7^, ^24^, ^59^ which limits our ability to make a direct comparison with other studies. Comprehensive subregional and global dGEMRIC analyses were not performed in our study, and it is possible that different associations may have been detected if other cartilage ROIs were sampled. However, the chondrolabral junction was deliberately chosen for this study as it is a specific region known to be prone to damage in FAI syndrome. Global measurement of dGEMRIC scores has the drawback that it is less sensitive in detecting abnormalities,^24^ and measurement of multiple subregions carries the statistical disadvantage of necessitating multiple tests of association, which increases the type I error rate. Two different 3 T MRI scanners were used for the acquisition of dGEMRIC scans in this study. Although statistical adjustment was made to correct for any systematic influence of the MRI scanner on the dGEMRIC score, there remains the possibility that this introduced heterogeneity into our results. As an exploratory study not designed for these analyses, insufficient statistical power is likely, and the possibility of type II errors must be acknowledged. Associations of more modest strength may exist with some of the variables tested, which are not detectable with the statistical power afforded by this study. There were particularly low numbers of people with high femoral, acetabular, and combined femoral and acetabular versions in this study, likely because this is more common in developmental dysplasia of the hip than FAI syndrome,^21^ and hence conclusions should not be drawn from this study about the effect of high version measurements on cartilage health. Further limitations of the study were its cross‐sectional nature, which precludes predictive or cause‐effect conclusions from being drawn, and the fact that the study's sample was drawn from participants in a clinical trial who may have characteristics different from those of the larger FAI syndrome population.

This study has clinical implications. Severe hip effusion–synovitis was associated with poorer cartilage health, which is of relevance to the pathogenesis of early osteoarthritis in FAI syndrome. Future research should investigate changes in cartilage health and synovitis longitudinally in FAI syndrome patients to determine if a causal relationship exists between synovitis and cartilage damage. If this were found to be the case, synovectomy or the various techniques for the management of cartilage lesions could be further investigated as potentially disease‐modifying treatments. As dGEMRIC is not often performed in the clinical setting, the presence of effusion–synovitis may also alert the clinician or radiologist to the possibility of early biochemical cartilage disease. Research is ongoing into newer, noncontrast MRI techniques for biochemical cartilage assessment such as T1ρ and T2*, which may eliminate the possibility of deleterious effects and/or adverse reactions from contrast.^36^, ^60^, ^61^ Low combined femoral and acetabular version was associated with maintained cartilage health at the chondrolabral junction. This highlights to the clinician the importance of assessing femoral and acetabular versions and considering their combined effect, on the propensity for impingement and damage to the hip joint. Future research may be directed at determining the optimal surgical solution for abnormalities of the femoral and acetabular versions, as at present this is controversial.

In conclusion, this cross‐sectional study found severe effusion–synovitis to be associated with poorer cartilage health in FAI syndrome, as measured by the dGEMRIC score at the chondrolabral junction. The lower combined femoral and acetabular version was associated with maintained cartilage health at the chondrolabral junction, which highlights the need to consider both femoral and acetabular versions together when assessing and treating FAI syndrome.

## AUTHOR CONTRIBUTIONS

Nicholas J. Murphy conceived the study, performed bony morphology and delayed gadolinium‐enhanced MRI of cartilage (dGEMRIC) measurements, and drafted the manuscript. Jillian Eyles and Libby Spiers performed data collection. Emily J. Davidson and James M. Linklater performed hip osteoarthritis MRI score analysis. Young Jo Kim performed dGEMRIC analysis. David J. Hunter conceived the study and oversaw the Australian FASHIoN trial. Jillian Eyles, Libby Spiers, James M. Linklater, Young Jo Kim, and David J. Hunter designed the Australian FASHIoN trial in collaboration with the UK FASHIoN trial leaders. All authors read and approved the final manuscript.

## CONFLICT OF INTEREST STATEMENT

David J. Hunter is a consultant to Pfizer, Lilly, TLCBio, and Merck Serono and is supported by an NHMRC Investigator Fellowship.

## Supporting information

Supporting information.

Supporting information.

Supporting information.

Supporting information.

Supporting information.
