# Supplementary material for: Combined femoral and acetabular version and synovitis are associated with dGEMRIC scores in people with femoroacetabular impingement (FAI) syndrome
Source: J Orthop Res. 2023 Apr 12;41(11):2484–94. doi: 10.1002/jor.25568 (PMC10946968; doi:10.1002/jor.25568)
Supplement: Supplementary file 1 — Supporting information. [file JOR-41-2484-s003.docx]

**Supplementary Table 1.** Hip Osteoarthritis MRI Score (HOAMS) intra- and inter-reliability results

|  |  | |
| --- | --- | --- |
| Joint feature | **HOAMS intra-observer agreement weighted kappa (95% CI)** | **HOAMS inter-observer agreement weighted kappa (95% CI)** |
| Cartilage | 0.671 (0.532, 0.809) | 0.843 (0.706, 0.981) |
| Bone Marrow Lesions | 0.563 (0.460, 0.667) | 0.771 (0.653, 0.888) |
| Subchondral Cysts | 0.656 (0.537, 0.774) | 0.565 (0.449, 0.681) |
| Osteophytes | 0.632 (0.467, 0.796) | 0.874 (0.676, 1.072) |
| Labrum | 0.836 (0.640, 1.031) | 0.725 (0.524, 0.927) |
| Synovitis | 0.529 (0.323, 0.734) | 0.372 (0.186, 0.558) |
| Dysplasia | 1.000 (0.494, 1.506) | 0.634 (0.163, 1.105) |
| Trochanteric bursitis | 0.222 (-0.096, 0.540) | 0.609 (0.143, 1.074) |
| Trochanteric insertional tendonitis | 0.667 (0.190, 1.144) | 0.400 (-0.106, 0.906) |
| Labral hypertrophy | 1.000 (0.494, 1.506) | 0.000 (0.000, 0.000) |
| Herniation pits | 1.000 (0.494, 1.506) | 0.700 (0.194, 1.206) |
| Paralabral cysts | 1.000 (0.494, 1.506) | 0.842 (0.342, 1.342) |
