# Supplementary material for: Combined femoral and acetabular version and synovitis are associated with dGEMRIC scores in people with femoroacetabular impingement (FAI) syndrome
Source: J Orthop Res. 2023 Apr 12;41(11):2484–94. doi: 10.1002/jor.25568 (PMC10946968; doi:10.1002/jor.25568)
Supplement: Supplementary file 2 — Supporting information. [file JOR-41-2484-s002.docx]

**Supplementary Table 2.** Univariate association of alpha angles measured in each radial plane with delayed gadolinium enhanced MRI (dGEMRIC) score*

|  | Beta coefficient | 95% CI | Partial correlation  (r_partial_) |  | P-value |
| --- | --- | --- | --- | --- | --- |
| Superior alpha angle (12 o’clock) | -1.43 | (-3.53, 0.67) | -0.21 |  | 0.176 |
| Superoanterior alpha angle (1 o’clock) | -0.34 | (-2.91, 2.24) | -0.04 |  | 0.793 |
| Anterosuperior alpha angle (2 o’clock) | -0.81 | (-3.87, 2.26) | -0.10 |  | 0.599 |
| Anterior alpha angle (3 o’clock) | -3.88 | (-6.49, -1.27) | -0.41 |  | 0.004 |
| Maximum alpha angle (all radial planes) | -2.15 | (-4.84, 0.58) | -0.23 |  | 0.121 |

*Adjusted for body mass index (BMI)
