# Supplementary material for: Combined femoral and acetabular version and synovitis are associated with dGEMRIC scores in people with femoroacetabular impingement (FAI) syndrome
Source: J Orthop Res. 2023 Apr 12;41(11):2484–94. doi: 10.1002/jor.25568 (PMC10946968; doi:10.1002/jor.25568)
Supplement: Supplementary file 3 — Supporting information. [file JOR-41-2484-s005.docx]

**Supplementary Table 3:** Pairwise comparison between different grades for Hip Osteoarthritis

MRI Scores (HOAMS) and Low, Normal and High version measurements, unadjusted for alpha

angle, with respect to delayed gadolinium enhanced MRI (dGEMRIC) score

|  | Mean difference ~(M_d_) | 95% CI of Md | Partial eta-squared | P-value |
| --- | --- | --- | --- | --- |
| Synovitis |  |  | **0.230** | **0.003** |
| Grade 0 – Grade 1 | -10.29 | -100.29, 79.71 |  | 1.000 |
| Grade 0 – Grade 2 | 172.77 | 33.72, 311.82 |  | 0.010 |
| Grade 1 – Grade 2 | 183.06 | 56.24, 309.88 |  | 0.002 |
| Subchondral cysts |  |  | **0.036** | **0.451** |
| Grade 0 – Grade 1 | -15.03 | -104.83, 74.77 |  | 1.000 |
| Grade 0 – Grade 2 | 78.81 | -99.70, 257.33 |  | 0.833 |
| Grade 1 – Grade 2 | 93.85 | -74.77, 104.83 |  | 0.629 |
| Labral injury |  |  | **0.037** | **0.653** |
| Grade 0 – Grade 1 | -13.41 | -409.71, 382.90 |  | 1.000 |
| Grade 0 – Grade 2 | 2.94 | -335.64, 341.53 |  | 1.000 |
| Grade 0 – Grade 3 | 71.77 | -302.25, 445.80 |  | 1.000 |
| Grade 1 – Grade 2 | 16.35 | -187.28, 219.98 |  | 1.000 |
| Grade 1 – Grade 3 | 85.18 | -152.62, 322.98 |  | 1.000 |
| Grade 2 – Grade 3 | 68.83 | -88.05, 225.71 |  | 1.000 |
| Bone marrow lesions |  |  | **0.026** | **0.558** |
| Grade 0 – Grade 1 | 113.62 | -185.94, 413.18 |  | 1.000 |
| Grade 0 – Grade 2 | 66.02 | -235.85, 367.90 |  | 1.000 |
| Grade 1 – Grade 2 | -47.60 | -472.13, 376.93 |  | 1.000 |
|  |  |  |  |  |
| Femoral version |  |  | **0.216** | **0.005** |
| Low (<10°) – Normal (10-25°) | 117.48 | 32.02 – 202.94 |  | 0.004 |
| Low (<10°) – High (>25°) | 38.89 | -80.66 – 158.43 |  | 1.000 |
| Normal (10-25°) – High (>25°) | -78.59 | -193.67 – 36.49 |  | 0.289 |
| Acetabular version |  |  | **0.126** | **0.051** |
| Low (<10°) – Normal (10-25°) | 81.91 | -71.50 – 235.32 |  | 0.572 |
| Low (<10°) – High (>25°) | 247.21 | 2.92 – 491.51 |  | 0.046 |
| Normal (10-25°) – High (>25°) | 165.30 | -36.15 – 366.76 |  | 0.141 |
| Combined femoral and acetabular version |  |  | **0.225** | **0.009** |
| Low (<20°) – Normal (20-50°) | 130.46 | 35.70 – 225.23 |  | 0.004 |
| Low (<20°) – High (>50°) | 17.73 | -184.10 – 219.55 |  | 0.999 |
| Normal (20-50°) – High (>50°) | -112.74 | -303.72 – 78.25 |  | 0.447 |

*Adjusted for body mass index (BMI), with Bonferroni adjustment for multiple comparisons
