# Supplementary material for: Combined femoral and acetabular version and synovitis are associated with dGEMRIC scores in people with femoroacetabular impingement (FAI) syndrome
Source: J Orthop Res. 2023 Apr 12;41(11):2484–94. doi: 10.1002/jor.25568 (PMC10946968; doi:10.1002/jor.25568)
Supplement: Supplementary file 4 — Supporting information. [file JOR-41-2484-s001.docx]

**Supplementary Table 4a:** Participants in each category for sensitivity analysis using different thresholds for ‘high’ version measurements

| **Femoral version, n (%):** |  | |  |
| --- | --- | --- | --- |
|  | | Low (<10°) | 17 (34%) |
|  | | Normal (10-20°) | 23 (46%) |
|  | | High (>20°) | 10 (20%) |
| **Acetabular version, n (%):** | |  |  |
|  | | Low (<10°) | 4 (8%) |
|  | | Normal (10-20°) | 36 (72%) |
|  | | High (>20°) | 10 (20%) |
| **Combined femoral and acetabular version, n (%):** | |  |  |
|  | | Low (<20°) | 11 (22%) |
|  | | Normal (20-40°) | 30 (60%) |
|  | | High (>40°) | 9 (18%) |
