# Supplementary material for: Combined femoral and acetabular version and synovitis are associated with dGEMRIC scores in people with femoroacetabular impingement (FAI) syndrome
Source: J Orthop Res. 2023 Apr 12;41(11):2484–94. doi: 10.1002/jor.25568 (PMC10946968; doi:10.1002/jor.25568)
Supplement: Supplementary file 5 — Supporting information. [file JOR-41-2484-s004.docx]

**Supplementary Table 4b:** Sensitivity analysis investigating the univariate association of version measurements with delayed gadolinium enhanced MRI (dGEMRIC) score, using different thresholds for 'high’ version measurements

|  | Mean difference ~(M_d_) | | 95% CI of M_d_ | Partial eta-squared | P-value |
| --- | --- | --- | --- | --- | --- |
| Femoral version | |  |  | **0.114** | **0.074** |
| Low (<10°) – Normal (10-20°) | 88.95 | | -12.23 – 190.13 |  | 0.102 |
| Low (<10°) – High (>20°) | 24.52 | | -87.79 – 136.83 |  | 1.000 |
| Normal (10-20°) – High (>20°) | -64.43 | | -164.10 – 35.24 |  | 0.344 |
| Acetabular version |  | |  | **0.062** | **0.250** |
| Low (<10°) – Normal (10-20°) | 55.55 | | -94.48 – 205.59 |  | 1.000 |
| Low (<10°) – High (>20°) | 106.00 | | -60.27 – 272.27 |  | 0.359 |
| Normal (10-20°) – High (>20°) | 50.45 | | -50.35 – 151.24 |  | 0.658 |
| Combined femoral and acetabular version |  | |  | **0.131** | **0.048** |
| Low (<20°) – Normal (20-40°) | 102.02 | | 2.00-202.05 |  | 0.044 |
| Low (<20°) – High (>40°) | 83.59 | | -37.19 – 204.36 |  | 0.276 |
| Normal (20-40°) – High (>40°) | -18.44 | | -117.51 – 80.64 |  | 1.000 |

*Adjusted for alpha angle and body mass index (BMI), with Bonferroni adjustment for multiple comparisons
